# Religiosity and Quality of Life in Older Christian Women in Ireland: A Mixed Methods Analysis

**DOI:** 10.1007/s10943-022-01519-3

**Published:** 2022-03-16

**Authors:** Joanna Orr, Rose Anne Kenny, Christine A. McGarrigle

**Affiliations:** 1 The Irish Longitudinal Study On Ageing (TILDA), Trinity College Dublin, 152-160 Pearse St, Dublin, Ireland; 2 Mercer’s Institute for Successful Ageing, St James’s Hospital, Dublin, Ireland

**Keywords:** Ageing, Quality of life, Religion, Mixed methods

## Abstract

This study used a mixed methods approach to understand the ways in which religion and quality of life (QoL) are associated in later life in Ireland. Longitudinal quantitative data from 2112 Christian women aged 57 and over at baseline (2009–10) participating in the Irish Longitudinal Study on Ageing (TILDA), as well as qualitative data from semi-structured interviews from 11 Christian women aged 65 and over in 2018, were used. The quantitative data showed an association between lower religiosity and lower QoL. Qualitative data supported an effect of religious involvement on QoL although certain aspects of being religious in Ireland were accompanied by distress. The data suggested that the relationship between religious attendance and higher QoL could be driven by multifactorial pathways, including psychological, social, and practical benefits.

**Supplementary Information:**

The online version contains supplementary material available at 10.1007/s10943-022-01519-3.

## Background

A majority of studies on the relationship between religion and well-being, positive emotions and happiness have reported positive associations, while a smaller number have presented mixed findings, often varying depending on the measure of religion or religiosity used (Koenig et al. 2012). There remains a need to uncover mechanisms behind the association between religiosity, quality of life, and mental health more generally. The integration of quantitative and qualitative methods to explore the link between religious practice and well-being has, to date, not been conducted.

### The Irish Context

The 20th Century in Ireland was a time of enormous social and economic change, even though this process of modernization lagged behind its closest European counterparts. A key delayed process of change was the decline of religious influence (Inglis, 1998). Throughout the century, Ireland remained one of the most religious countries in Europe, and the most religious country in Western Europe. However, religious belief and practice are currently in decline. The proportion of those reporting no religion has grown at each census since 1971, while the proportion of Catholics, the majority religious denomination, has declined from 94.9% in 1961 to 78.3% in 2016 (Central Statistics Office, 2017).

The role of the Church in Irish society cannot be overstated. The Catholic Church was directly involved in running education, health care and other social provisions. Catholic ethos influenced marriage, childrearing and women’s role in society (Inglis, 1998). Both coming of age in a homogenous religious context, as well as experiencing a seismic change in social values, values which probably shaped one’s own moral compass and worldview, could potentially have impacts on a person’s well-being. Further, the decline of the Catholic Church in Ireland has been marked by abuse scandals, which have the potential of making this decline more painful for those who value the Church’s role in their lives. Evidence suggests that religious attendance has declined in the population as a direct result of Church abuse. Further, for victims of Church abuse, loss of faith was common (Goode et al., 2003).

### Quality of Life

QoL is a concept which encompasses numerous positive human emotions and circumstances. Various definitions and measurements exist of quality of life; In the Irish Longitudinal Study on Ageing (TILDA), QoL is measured using the CASP-19 scale and, in recent waves, the shorter CASP-12 scale (Hyde et al., 2003; Sexton et al., 2013). The CASP-19 scale was developed for use in older people and goes beyond the satisfaction of basic needs, to include higher needs (mastery, creativity, etc.) (Hyde et al., 2003; Maslow, 1968; McKenna et al., 1999). The CASP-19 was devised using four domains of need, which are treated as equal and inseparable. These domains are Control, or the ability to control one’s environment; Autonomy, or the freedom from the unwanted interference from others; Self-realization; and Pleasure. These domains have been found to form a single latent QoL factor (Hyde et al., 2003).

Previous analysis from TILDA has found that in the over 50 s, QoL tends to increase and then peak around age 67, declining thereafter (Ward et al., 2019). As QoL is a multidimensional construct, it is influenced by social, economic and health determinants. Health declines have direct impacts on QoL (Sexton et al., 2015). Health deteriorations encountered by most in later life mean that the older population are vulnerable to declining well-being. Other life domains which are linked to QoL include mental health, personality, genetics, social activity, education and income (Koenig, King, and Carson 2012; Ward et al., 2019).

### Religion and QoL

A review of the relationship between religion and QoL found that 78% of studies published between 2000 and 2010 reported positive associations between religion and QoL (Koenig et al., 2012). A more recent review outlined how religion can help fulfil the higher order needs required for well-being at the individual level, such as purpose and meaning, social support, and coping strategies (Tay et al., 2014). It also found that the associations between religion and well-being hold across cultural contexts, although they are strongest in more religious countries. This assessment is partially supported by a large-scale analysis of cross-sectional, multi-country data for the years 2005–2011 (Graham & Crown, 2014). This analysis suggested that religious attendance was associated with hedonic happiness and evaluative satisfaction with life, while religious importance was only associated with hedonic happiness. The worldwide data presented gave rise to questions on a perceived paradox; although the religious reported higher well-being, countries where the majority were religious had lower well-being overall (Deaton & Stone, 2013; Myers & Diener, 2018).

Recent work has shown that in countries where the state was responsible for important aspects of QoL, such as health and education, religion was no longer as strong a predictor of QoL (Zuckerman et al., 2018). This suggests that religion is, at least in part, a means for fulfilling certain needs, in particular in contexts where these are difficult to attain in other ways (Diener et al., 2011; Graham & Crown, 2014). A review of the current state of the science on happiness concludes “it appears that religiosity is a frequent but not a universal predictor of higher SWB [subjective well-being], and its effects and mediators depend to some degree on culture and the life circumstances of respondents” (Diener et al., 2018).

## Methods

**Fig. 1:**
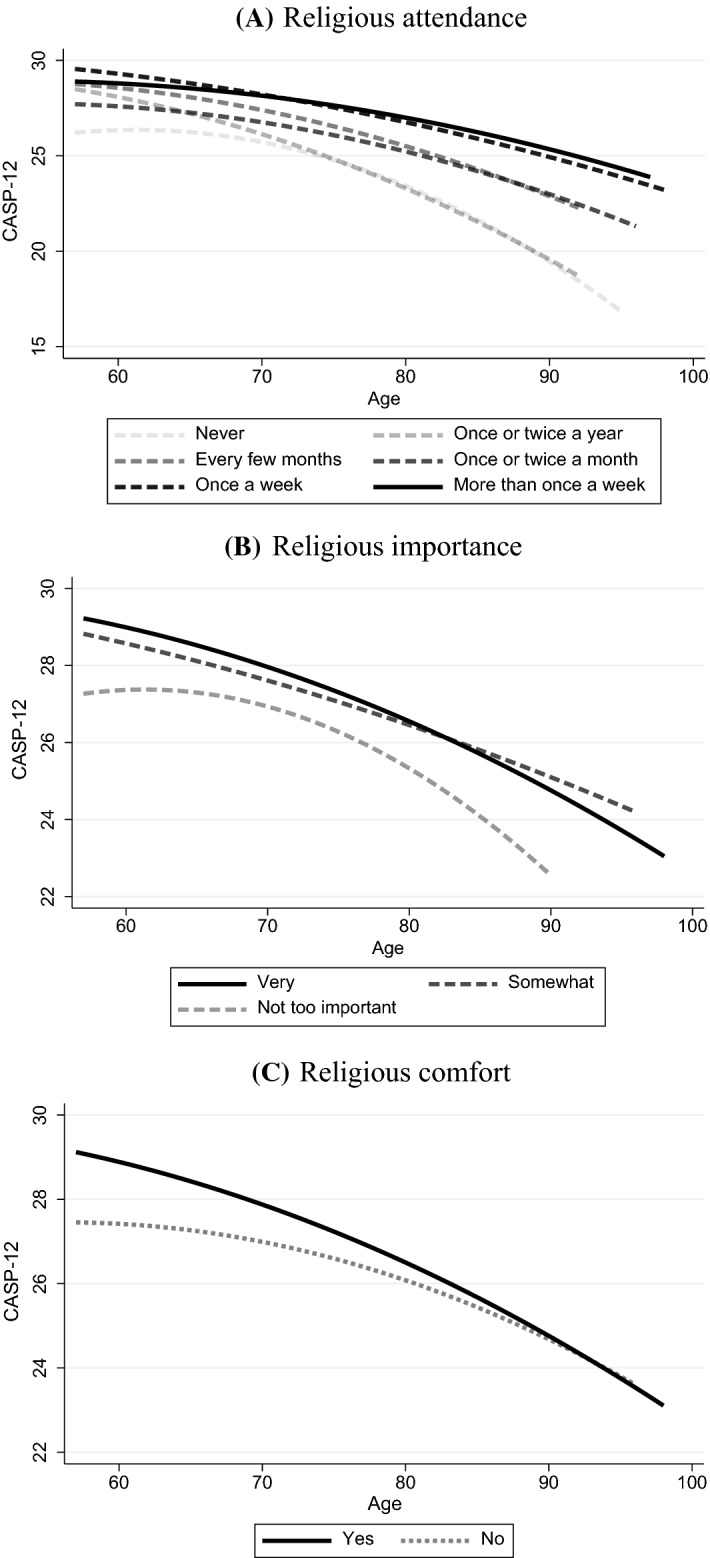
Plotted predicted CASP − 12 by religious attendance (**A**), Importance (**B**) and Comfort (**C**)

We used a convergent mixed methods approach (Creswell & Clark, 2010) to compare the results from a longitudinal analysis of a large nationally representative study of adults aged 50 and over with the results of a small qualitative study on religious participation and practice in older Irish women aged 65 and over. We used quantitative data to investigate whether QoL differs by level of religious attendance, religious importance, and religious comfort and strength. Qualitative data were used to explore the ways in which religious engagement throughout the life-course and in later life helps shape women’s well-being. The mixed method analysis examined whether contexts and narratives from the qualitative data help to provide a deeper understanding of the patterns and consequences of religious attendance on well-being seen in the quantitative data (Fig. 1).

### Quantitative Data

TILDA is a longitudinal, nationally representative study of the over 50 s population in Ireland. Our study used data from the first five waves of TILDA, completed between 2009 and 2018. Respondents were interviewed using a computer-assisted personal interview (CAPI), as well as a pen and paper self-completion questionnaire (SCQ). Ethical approval for each wave of TILDA was granted by the Trinity College Dublin Faculty of Health Sciences Research Ethics Committee, and informed consent was obtained. Detailed descriptions of the TILDA methodology are given elsewhere (Donoghue et al., 2018; Whelan & Savva, 2013). For comparability with the qualitative data, we focused on Christian women (including Catholic, Church of Ireland and other Christian) aged 57 years and over. Women aged 57 or over at baseline in TILDA (2009–10) match the age of the qualitative sample (65 at interview in 2018).

QoL was measured in TILDA using the Control, Autonomy, Self-actualization and Pleasure scale (CASP) (Hyde et al., 2003). Waves 1 and 2 of TILDA used the full CASP scale, which includes 19 items (CASP-19). Subsequent waves have used a shorter 12 item scale, validated for use with TILDA data (Sexton et al., 2013). Data from waves 1 and 2 were recoded to include only the CASP-12 items, and this is the measure used in the current analyses. Each item in the scale is given a score between 0 and 3, with the total scale ranging from 0 to 36. Higher scores denote higher quality of life. CASP items were collected at each wave as part of the Self-Completion Questionnaire (SCQ) which was completed and returned by post. The SCQ had a lower response rate at each wave than the CAPI interview, therefore, the sample for participants with CASP data is smaller than the main sample. SCQ response rates were 85% at Waves 1, 2 and 3, and 86% at Waves 4 and 5.

To examine whether religiosity can impact QoL, we examined three religiosity variables. These were religious attendance (“About how often do you attend religious services?” Never/almost never; Once or twice a year; Every few months; Once or twice a month; Once a week; More than once a week); religious importance (“How important would you say religion is in your life?” Very important; Somewhat important; Not too important/Not at all important); and religious comfort and strength (“Do you find that you get comfort and strength from religion or not?” Yes; No). We chose to use the responses denoting the ‘most’ religious as our reference categories: those who attended religious services more than once a week, those who said religion was very important to them, and those who derived comfort and strength from religion.

We analysed the first five waves of TILDA by fitting linear mixed effects models of the trajectories of CASP-12 over time. Random slopes were allowed for age, and models were adjusted for covariates. We used unstructured random effects covariance which allows for variances and covariances between all variables and does not assume a covariance structure (StataCorp, 2013). We included religiosity measures individually and included interactions between religiosity factors and age and age^2^ to assess whether CASP-12 score trajectories varied over time by religiosity. Fitted predicted scores were plotted for each model by religiosity to aid the interpretation of results. Model fit and collinearity were assessed.

Covariates were chosen according to the religiosity and QoL research literature. Religiosity in Ireland has been found to be higher in older adults, women and those with lower levels of education (O’Mahony, 2011; Breen 2014). Recent work from TILDA also found that age, gender and education, among other socioeconomic characteristics, had an important role to play in QoL (Ward et al., 2019). All models were initially adjusted for age only and subsequently adjusted for socio-demographic characteristics (education, marital status, home ownership), social network and social participation (number of close family and friends, participation in any social or sports group) and health and disability characteristics (limitations in activities of daily living (ADL), limitations in instrumental activities of daily living (IADL), self-rated health, self-rated mental health, fear of falling, chronic pain and incontinence).

### Qualitative Data

Qualitative data were collected with the aim to gain an understanding of the experiences of women living in Ireland and their health, well-being and religious participation in the context of getting older. The study used a convenience sampling strategy to recruit women aged 65 and over from various church congregations in Dublin. Administrators of 14 churches (11 Catholic, two Church of Ireland, one Presbyterian) were contacted seeking permission to advertise to the congregation. Seven of these churches ran recruiting advertisements for the study, through notices in church newsletters or recruitment posters on church noticeboards. Nine participants were recruited from five churches. Two of these participants were friends and contacted the study together but were interviewed individually. A further two participants were recruited into the study by friends who were congregants of the targeted churches. The final sample of 11 women were aged between 67 and 89 and lived in Dublin, except for one respondent who travelled from another Irish city to participate. Details of participant age, marital status and religious affiliation are given in Supplemental Table A. All participants gave informed consent to participate in the study, and the study received ethical approval from the Trinity College Dublin School of Medicine Research Ethics Committee.

Each participant completed an individual semi-structured interview with the same researcher. The interview schedule used is presented in Supplemental Table B. Interviews lasted between 50 min and two hours and were conducted in Trinity College Dublin, apart from one which was conducted in the participant’s home. The interview instrument was developed to explore the women’s experiences of health, ageing, and religion in Ireland, but was also flexible enough to allow for further themes to emerge. Predetermined themes included trajectories of religious engagement, religious practices, personal beliefs, community participation, family/networks, and health and well-being. All interviews were conducted, recorded, and subsequently transcribed by the same interviewer.

Thematic analysis was used; this generally consists of five interconnected and nonlinear phases: compiling, disassembling, assembling, interpreting and concluding (Castleberry & Nolen, 2018; Yin, 2010). The analysis process was begun during interviewing and transcribing, throughout which memos were taken. Data were initially disassembled, or coded, using predetermined themes. Flexibility in the data collection instrument and process of analysis allowed for new themes to be generated from the data. This phase of analysis requires analytical and reflective thinking by the researcher. The researcher was guided by a set of questions set out by Cooper and colleagues (Castleberry & Nolen, 2018; Cooper & Camic, 2012) to query and review the thematic hierarchy developed for the data. These include “Is this a theme, and if so, does it inform my understanding of the research question?” “Does the data sufficiently support this theme?” and “Is this theme coherent?” Interpretation of the data was conducted at each stage of data analysis, with final interpretation consisting of drawing conclusions based on the wide themes within the data, as well as more granular insights. Data analysis was conducted in NVivo 12 by the same researcher, and intra-rater reliability was used to check coding consistency. Pseudonyms were used to maintain anonymity in reported results.

## Results

### Quantitative Data

Data were available for a total of 2,112 TILDA participants aged 57 and over at baseline. Descriptive statistics for religious factors, CASP-12 and covariates are given in Table 1. Most of the sample reported attending religious services regularly, with half attending once a week (50.5%). Over half reported that religion was very important to them (61.7%), although 12.2% said religion was not important to them. A large majority reported deriving comfort and strength from religion (85.8%). CASP-12 scores at baseline had a mean of 28.5 (SD = 5.1).

**Table 1:** Characteristics of the sample of Christian women aged 57 and over (*n* = 2,112)

Measures	Total %/Mean (SD)
*Age (mean (SD))*	66.7 (7.4)
*CASP-12 Wave 1 (mean (SD))*	28.5 (5.1)
*Religious attendance %*	
Never/almost never	7.1
Once or twice a year	7.5
Every few months	6.9
Once or twice a month	6.7
Once a week	50.5
More than once a week	21.2
*Religious importance %*	
Very important	61.7
Somewhat important	26.1
Not very/Not at all important	12.2
*Religious comfort %*	
Yes	85.8
*Education %*	
Primary/None	27.0
Secondary	40.8
Third level/Higher	32.2
*Home ownership %*	
Owns outright	85.6
Owns with mortgage	8.0
Renting	6.4
*Self-rated health %*	
Excellent	15.6
Very good	28.4
Good	34.2
Fair	17.4
Poor	4.4
*Marital status %*	
Married	64.0
Never married	7.7
Separated/Divorced	6.4
Widowed	21.8
Chronic pain %	40.5
Incontinence (monthly or more frequent) %	13.6
Fair/poor self-rated mental health %	9.0
Fear of falling %	32.4
Participation in social or sports groups %	53.1
ADLs (mean (SD))	0.1 (0.5)
IADLs (mean (SD))	0.1 (0.5)
Close relatives / friends (mean (SD))	10.8 (6.0)

CASP-12 scores are presented for Wave 1 only. Not all respondents had CASP-12 data at Wave 1. CASP-12 data for all waves is presented in Supplemental Table C

Table 2 shows results for linear mixed effects models of the relationship of religious attendance and CASP-12. Model 1 includes religious attendance at baseline and religious attendance with age interactions. The model showed that, compared to those who attended more than once a week, those who never attended had CASP-12 scores which were 2.8 points lower on average, or over half of an SD. Model 2 was adjusted for covariates and showed that only those attending religious services once or twice a year or once or twice a month maintained significantly lower CASP-12 scores. Age interactions were not significant.

**Table 2:** Fixed and random effects models; religious attendance and CASP-12, and interactions with time (*n* = 2,043)

Measures	Model 1. Religious attendance only		Model 2. Covariate adjusted	
Fixed effects	Coef	95% CI	Coef	95% CI
Age centred	− .12***	− .18, − .06	− .11***	− .16, − .06
Age centred squared	− .00*	− .01, − .00	− .00*	− .01, − .00
*Religious attendance (ref: More than once a week)*				
Once a week	− .14	− .72, .43	.09	− .39, .57
Once or twice a month	− 1.75***	− 2.69, − .81	− .92*	− 1.70, − .15
Every few months	− .98*	− 1.91, − .04	− .22	− 1.00, .56
Once or twice a year	− 2.02***	− 2.92, − 1.12	− 1.25**	− 2.01, − .50
Never	− 2.79***	− 3.73, − 1.86	− .75†	− 1.54, .04
Constant	28.76***	28.27, 29.24	28.92***	27.92, 29.91
*Random effects*				
Variance (age)	.00	.00, .10	.00	.00, .04
Variance (constant)	17.43	16.15, 18.81	10.25	9.41, 11.16
Covariance (age, constant)	− .04	− .12, .05	.06	.00, .13
Residual variance	9.00	8.65, 9.37	9.00	8.66, 9.36

*p < 0.05, **p < 0.01, ***p < 0.001, †*p* < 0.1

Interaction effects not shown

Results for religious importance are given in Table 3. Those who reported religion was not important to them had CASP-12 scores which were 1.2 points lower on average when compared to those who reported religion was very important to them in an unadjusted model. When adjusting for covariates, the effect of religious importance diminished but remained significant. Neither model showed significant age interactions. Religious comfort models are shown in Table 4. Reporting comfort and strength from religion was associated with higher CASP-12 scores in an unadjusted and adjusted model, although the effect size declined after adjustment. Again, no age interactions were significant.

**Table 3:** Fixed and random effects models; religious importance and CASP − 12, and interactions with Time (*n* = 2,039)

Measures	Model 1. Religious importance only		Model 2. Covariate adjusted	
Fixed effects	Coef	95% CI	Coef	95% CI
Age centred	− .11***	− .14, − .08	− .10***	− .13, − .07
Age centred squared	− .00***	− .01, − .00	− .00**	− .00, − .00
*Religious importance (ref: Very)*				
Somewhat important	− .39	− .90, .11	− .22	− .63, .19
Not too important	− 1.21***	− 1.88, − .54	− .71*	− 1.27, − .16
Constant	28.39 ***	28.11, 28.67	28.89***	27.96, 29.82
*Random effects*				
Variance (age)	.00	.00, .05	.00	.00, .03
Variance (constant)	18.04	16.72, 19.47	10.38	9.53, 11.30
Covariance (age, constant)	− .03	− .12, .06	.06	.00, .13
Residual variance	9.01	8.65, 9.37	9.00	8.65, 9.36

*p < 0.05, **p < 0.01, ***p < 0.001, †*p* < 0.1

Interaction effects not shown

**Table 4:** Fixed and random effects models; religious comfort and strength and CASP − 12, and interactions with time (*n* = 2,014)

Measures	Model 1. Religious importance only		Model 2. Covariate adjusted	
Fixed effects	Coef	95% CI	Coef	95% CI
Age centred	− .09**	− .14, − .04	− .09***	− .15, − .04
Age centred squared	− .00†	− .01, .00	− .01*	− .01, − .00
Religious comfort	1.02**	.41, 1.62	.58*	.08, 1.07
Constant	27.28 ***	26.72, 27.83	28.28***	27.29, 29.27
*Random effects*				
Variance (age)	.01	.00, .04	.01	.00, .03
Variance (constant)	17.85	16.52, 19.27	10.28	9.43, 11.20
Covariance (age, constant)	− .03	− .12, .05	.06	− .00, .12
Residual variance	8.97	8.62, 9.34	8.98	8.64, 9.34

*p < 0.05, **p < 0.01, ***p < 0.001, †*p* < 0.1

Interaction effects not shown

### Qualitative Data

#### Religious Practice as a Source of Well-being

In-depth interviews uncovered various ways in which religion was a facilitator for QoL. All the women interviewed mentioned some way in which they felt religion was associated with spiritual and mental well-being. Most respondents (n = 8) mentioned ways in which religious practice or belief helped them or others cope with adverse life circumstances, which included physical health crises, death or illness of loved ones, and mental health difficulties.

“I’d go back and I’d plod along again, and the only thing, I’d go to mass and I’d say, Lord, give me strength. To just keep going. And I’d get the strength, and I’d get up and I’d go again.”

Fiona, 67

Margaret’s experience of returning to her faith later in life was an example of turning to religious practice when confronted with difficult circumstances.

“I was 50 years of age and I went back [to the church] in earnest. I had a big operation. And I had problems breathing […] And then I started praying to God, and telling God that I’d be back for sure, and I’d never miss again. That’s when I was 50, that was 30 years ago. So, I really made up for it, then.”

Margaret, 80

Three respondents also discussed spiritual and religious feelings and practice which led to inner peace, calm and tranquillity. These feelings were for the most part associated with religious attendance, although they were also related to attributing religious feeling to other events in their lives.

“It’s peaceful and it flows over me. It’s like music. It flows over you and you absorb some but not others, you know? Suddenly mass would be over and I’d think oh my God I didn’t say a prayer all through that mass. But those things happen.”

Geraldine, 81

Only one respondent explicitly mentioned religion as a source of meaning and purpose in her life. However, this was a strong theme for this respondent, and she mentioned it as both important for her own well-being, and that of others.

“I would think that my religious beliefs have given me a better quality of life, than I would have if I didn’t believe. Because, as I said to you earlier, they have given me, my beliefs give me a purpose and a meaning.”

Kathleen, 69

#### Community Engagement

Most of the women (*n* = 9) spoke about the role of religion in maintaining engagement and activity as part of the community, in particular for older people. Some, like Anne, acknowledged that religion was an important source of mental and spiritual well-being for others, but believed it impacted on her personally through more active and social mechanisms. This passage from Anne highlights that, even though some may not experience feelings of religious or spiritual well-being, there are other ways in which they gain from religion. Anne’s religion manifested through active participation in her community, instead of meditation, prayer, or other forms of individual religious practice.

“Now, I’m useless at meditation, I know this, I have tried it a few times, not for me […] Again, my version of religion is to be helping people, doing things. Not sitting in a church on your own, pretending to have a conversation with God. […] I mean there are people who are very devout, and I don’t knock that, you know, and they get tremendous, erm, kind of, self-fulfilment from it…”

Anne, 71

Later in her interview, Anne mentioned that religion is useful for ‘putting her in the way of opportunities’. Other interviews highlighted how community resources may not come directly from the church, but access to them was facilitated through church engagement. Examples of these include social clubs such as ladies’ clubs or active retirement clubs and resources such as meals on wheels. These were not directly organized by the church, but borrowed church space, were comprised of church parishioners and volunteers, and advertised within the church. Mary’s experience, below, of joining a religious youth organization when moving to Dublin is an example of some of the resources available to religious youth.

“I joined the Legion of Mary when I came to Dublin. That’s what we all did, it was the norm as well. And we had a great social life and everything, as well as help in doing a bit of work.”

Mary, 81

The sense of belonging to a community which can be gained from religious engagement was summarized by an anecdote from Eileen.

“Well, when I was at the bus stop today coming in, and a girl says to me, oh Mrs […], I haven’t seen you in ages, were you sick?”

Eileen, 85

Religious engagement can provide a point of contact with others in the community. For older people, in particular those living alone, religious practice which is routine can be helpful in creating a social network. As well as plausible psychological benefits from this type of social participation, possible practical benefits include a ‘safety net’ of people who may notice if an older member of the congregation was unwell or not present and may provide practical support if needed.

#### Religion as a Source of Conflict

The interviews also showed that religious engagement and social conflict related to religion could be associated with negative experiences. For many of the women (n = 8), religious practices became a point of contention with younger generations. Anne struggled with her daughter’s decision not to have her son receive his first communion, an important sacrament for Catholic children.

“And she [Anne’s daughter] said no, she wasn’t going to be a hypocrite. And I got very upset. I thought, you know? It’s a bit like going to university. I said in the moment, you’re missing a link here. And I get upset when I think about it. I kind of… but I can’t live someone else’s life. […] And… what I, what I do now is I… I avoid like the erm, first communions and Sunday masses, I don’t go near the church. It’s too upsetting. And I probably won’t for a long time.”

Anne, 71.

The very normative nature of religiosity during the women’s early years made societal changes in religiosity more difficult. For some, there was a feeling of losing a majority place in society.

“Catholics are definitely discriminated against in the media. […] Now, I think were actually being discriminated against, for being Catholics.”

Teresa, 89

Teresa’s feelings of being discriminated against reflect the perceived shift from having a majority position in society to a less privileged one. Others expressed feelings of exclusion:

“Ah, yes, you say a prayer at all, you’re a holy Joe. Ah yeah, yeah. No laugh, no fun at all, holy Joe.”

Patricia, 82

Although many would comment on the change in religious practice and belief in the country, and frame it as a negative, this did not appear to have a substantial impact on the well-being of the majority. However, the Church’s influence over women’s reproductive and family choices was felt by one respondent to have been an intrusion on her life.

“ was very angry with the Church about, you know, I have five children. My husband just said, God, he got told to increase and multiply, but he didn’t say by how many. He thought the ideal family would be two children. But anyhow, we ended up with [more]. Now I wouldn’t give any of them back, but, it just was, it made life very difficult. And you know, then it’s not fair to the children either.”

Geraldine, 81.

Finally, one respondent was affected by Church abuse scandals personally, and the effect on her self-esteem and happiness was profound. Brigid discussed finding out that a priest who she had been close with and greatly admired had been implicated in sexual abuse.

“And that, that… all those happy [memories]… I wouldn’t, even if my husband was alive and in very good health, I wouldn’t have told him. He wouldn’t know, you know, he wouldn’t know about it. But, being terrible disappointment and upset for me. […] So they’re the kind of things, it dents your faith you see, when you see things gone on.”

Brigid, 74

## Discussion

Quantitative data from TILDA showed that women aged 57 and over in Ireland had good QoL on average and were likely to be regular Church attenders, as well as considering religion to be important and finding comfort and strength in religion. Mixed effects models showed that higher religious attendance, importance and comfort were associated with higher QoL scores, but that these effects were small and diminished substantially after adjustment for covariates. There were also no interactions between religious measures and age, suggesting any association between QoL and religiosity was already established at the baseline observation at age 57 and over.

Qualitative data illustrated some of the ways in which religious practice, belief and belonging may impact well-being, and that this association was not always entirely positive. The women described religious practice, in particular, as being conducive to well-being. Meditative and calming effects were associated with church attendance and religious practice for many in the sample. For others, belonging to a religious community provided opportunities to develop and strengthen community ties, as well as access to community resources and activities. However, inter-generational conflict also emerged as a theme, where many of the women felt that their religious beliefs put them at odds with younger family members, as well as modern Ireland.

The integration of quantitative findings, showing slightly higher QoL in the more religious, and qualitative findings, showing the ways in which religion can be supportive of well-being, strengthens the validity of both results. In particular, our findings suggest that religious attendance has the most consistent effect on QoL, and that the differences between high and low attenders observed in the quantitative analysis arise from religious practice as a source of inner peace and tranquillity, as well as the way habitual religious behaviour can be a source of opportunities, all of which are conducive to health. The fact that religious practice does not seem to influence the change in CASP-12 scores over time suggests that the level of well-being afforded by religious practice is already set earlier in the life course. If there is a protective effect of religious practice, it may be established and practiced from an early age.

The qualitative data also add a new dimension to the quantitative results; we see that the change in the role of the Catholic church in Ireland in the women’s lifetimes has been difficult for many of them. The feeling that the religious context was changing around them was evident in many narratives, in particular when discussing relationships with younger family members, or the religious change in modern Ireland. There was also evidence of an internal conflict with the Church for some of the respondents; Church abuse had direct and indirect impacts on their feelings about the Catholic Church. For some, like Brigid, the impact was strong and direct. But for others, the influence of the Church on family-formation decisions was a point of resentment many years later.

Evidence from other studies supports some of our findings. Previous research has shown that religious attendance is associated with lower depressive symptoms in older Irish adults (Orr et al., 2019). The religion and well-being literature has also often looked at whether religion can help buffer the negative effects of illness. Europe-wide data have provided evidence of an effect of religious education; Ahrenfeldt and colleagues found that much of the variance in health outcomes for the religious could be explained by religious education; those who were religiously educated fared better, and those who were religiously educated and took part in religious organization had the best health outcomes (Ahrenfeldt et al., 2017). They make the argument that this sets up individuals to have religiosity which is either ‘restful’ or ‘crisis’, of which restful religiosity is beneficial to better mental health. This mirrors Pargament’s early ideas on negative and positive religious coping (Pargament et al., 1998). The observations within our data fit within these frameworks. The quantitative data show that, while there is an association between religious attendance and QoL, this appears to be set by age 57 and does not influence the change in QoL over time from this age onwards. The women’s own experiences show that, for many, religion was constant and habitual, although others did experience ‘crisis religiosity’.

Another important insight afforded by the qualitative data was insight into a society in which religious values are changing. There was a range of experiences of changing societal attitudes towards religion, from defiance, feelings of exclusion and betrayal. These narratives reflect the wider social change which Ireland has been undergoing in the women’s lifetimes. It is plausible that this social change, and the decline in the social position of the Church, may have left many of these women in a less stable social position. Religiosity as a social value has been declining, and this has the potential to impact on the culture-person match for religious people (Gebauer et al., 2017). There is evidence that religious people are happier in religious countries (Stavrova et al., 2013). Graham and colleagues’ analysis of an international dataset only found two exceptions to this rule: indigenous and Druze religions were not happier than their counterparts (Graham & Crown, 2014). This was not discussed in the paper by the authors but could be reflecting a minority position of these religious groups within their wider societies. In Ireland, as the country becomes less religious, this may plausibly have an impact on the well-being of those who are religious.

This study has some limitations; the findings specifically relate to women of Christian, mainly Catholic, faith in Ireland. They are not generalizable to other religious contexts. We did not interview women who have experienced Church institutional abuse in Ireland and so we are not able examine how religious practice or belief may be associated with the QoL of this vulnerable group. The number of participants in our qualitative study was also small, and the sampling strategy is likely to have biased our sample towards gregarious women willing to talk about their experiences. Our sample may have also been biased by the fact that only five of the churches approached participated in recruitment; this may be indicative of a more open and community-minded culture in the congregations themselves. Our study also has many strengths; the use of longitudinal, nationally representative data, which includes several health and social measures, has allowed us to accurately assess the independent association between religious attendance and QoL. In addition, the combination of this with in-depth interviews with women provided a unique perspective on the life experience and motivations surrounding religious practice and belief of women in Ireland.

## Conclusion

We find evidence that those with religious practice in the form of frequent religious attendance have better QoL. Qualitative evidence suggests this may be related to the potential for religious practice to provide feelings of calm and tranquillity for some individuals. Religious practice may also provide opportunities for social engagement and activities, as well facilitating access to resources. We did find some evidence that in some circumstances, religiosity could be a source of conflict, suggesting that the relationship between religion and QoL is not necessarily linear. The quantitative data showed an overall association between higher religiosity and higher QoL. Although the associations were attenuated when adjusting for covariates, they remained significant. Interactions with age were not significant, suggesting the association was already established when the women were first observed, and that it did not change as they became older. This association between religiosity and higher QoL provides incentive for ensuring that older adults have the support to maintain their religious practices as desired.

## Supplementary Information

Below is the link to the electronic supplementary material.

Supplementary file1 (DOCX 18 kb)

## Declarations

### Data Availability

TILDA data is available through University College Dublin’s Irish Social Science Data Archive (ISSDA)—https://www.ucd.ie/issda/data/tilda/
