# Supplementary material for: Religiosity and Quality of Life in Older Christian Women in Ireland: A Mixed Methods Analysis
Source: J Relig Health. 2022 Mar 16;61(4):2927–44. doi: 10.1007/s10943-022-01519-3 (PMC9314271; doi:10.1007/s10943-022-01519-3)
Supplement: Supplementary file 1 — Supplementary file1 (DOCX 18 kb) [file 10943_2022_1519_MOESM1_ESM.docx]

**Supplemental Material**

**Supplemental Table A**

*Qualitative study participant characteristics.*

| Participant pseudonym | Age | Marital status | Affiliation |
| --- | --- | --- | --- |
| Fiona | 67 | Divorced | Catholic |
| Bernie | 77 | Married | Catholic |
| Teresa | 89 | Widowed | Catholic |
| Anne | 71 | Married | Catholic |
| Kathleen | 69 | Never married | Catholic |
| Eileen | 85 | Married | Catholic |
| Brigid | 74 | Widowed | Catholic |
| Patricia | 82 | Never married | Catholic |
| Mary | 81 | Never married | Catholic |
| Geraldine | 81 | Widowed | Catholic |
| Margaret | 80 | Widowed | Church of Ireland (Anglican) |

**Supplemental Table B.**

| **Central Aim: To explore how women relate religious practice, belief and participation to their wellbeing in later life.** | |
| --- | --- |
| **Topics** | **Questions** |
| **Intro** | **Maybe to start off you could tell me a little bit about your life at the moment?**  May I ask what age you are now?  How would you identify your religious affiliation?  Where do you live?  Do you enjoy living in…?  Are you involved with any religious congregation in…?  Do you attend religious services anywhere else? |
| **Life course trajectories of engagement with religion** | *As we’ve discussed, this research wants to hear about your experiences with ageing and your engagement with religion. I would now like to ask you some questions about your religious engagement and beliefs throughout your life.*  **Could you tell me a little about your life growing up?**  *Prompt:*  Where did you grow up?  What was growing up in … like?  Was your family religious?  What was your relationship with your family like?  **Have you always considered yourself to be a religious person?** |
| **Religious practices** | **Could you tell me a little bit about your religious practices?**  *Prompt:*  For example, do you regularly go to church?  Or do you practice religion more privately?  Or in what other ways do you practice your religion?  Would you say you engage with your religion every day, or?  **And would you say it is necessary to practice religion in a certain way, or that there is room for interpretation in the conduct of one’s religious practice?** |
| **Personal beliefs** | **Could you tell me what religion / your faith means to you personally?**  *Prompt:*  What does it mean to you to be religious?  How would you describe your relationship with God / your faith?  **Have you always felt this way about your faith?**  *Prompt:*  Where there any times in your life when you felt differently?  **What has shaped the way you think about your faith throughout your life?**  *Prompt:*  Are church teachings important to you? Or do you find your own thinking and interpretation of your faith more important?  **And have you ever felt that your personal religious beliefs affect your views on social issues?**  *Prompt:*  Have you relied on religious teachings or Church statements to decide on how you feel about certain issues? |
| **Community and participation** | **Can you tell me about the community involved in [your local religious organization]?**  *Prompt:*  What are the people in the congregation / community like?  Is it a welcoming environment?  Do you feel a part of the community?  **And are there members of the congregation who you feel closer to?**  *Prompt:*  Do you ever spend time with members of the congregation outside of church? What kinds of things do you do?  Would you feel comfortable getting in touch with anyone from church if you needed help or support with anything? Has anything like this ever happened? |
| **Family and other social networks** | *Now if you don’t mind I’d like to find out a little bit more about other relationships in your life.*  **Who do you currently live with?**  **Do you have any children?**  **Do you have any other family who are close to you?**  **Is religion an important part of family life for you?**  **Has religion always been an important part of family life for you?**  **Do you have any family or friends who are not religious? What is this relationship like?**  *Prompt:*  Does it matter to you that they are not religious?  Does it affect the quality of the relationship in any way? |
| **Health and wellbeing** | *Now I’d like to talk about how you feel in terms of your health and happiness.*  **Could you tell me about what being [age] has been like for you?**  *Prompt:*  Do you feel your age?  How is your health generally?  How has getting older affected you?  **And do you think being religious has an effect on your wellbeing? Could you tell me about that?**  *Prompt:*  What aspects of your faith or religious practice do you think could be good for your health/wellbeing?  **How would your life be different if you were not able to practice?** |
| **Closing** | **Is there anything else you which we have not discussed and that you would like to add?**  **Debrief:**   - Would you like me to keep you updated on results or any published work that may come out of this? - What would be the best way to keep you updated? - The information sheet I gave you has all my information in case you need to get in touch with me with any questions or queries. |

**Supplemental Table C.**

*CASP-12 scores at each wavefor sample of Christian (Catholic or other) women aged 57 and over (n=2,112)*

|  | Total |
| --- | --- |
|  | Mean (SD) |
| CASP-12 Wave 1 | 28.5 (5.1)  (n=1,822) |
| CASP-12 Wave 2 | 27.5 (5.3)  (n=1,854) |
| CASP-12 Wave 3 | 27.2 (5.3)  (n=1,670) |
| CASP-12 Wave 4 | 27.7 (5.4)  (n=1,478) |
| CASP-12 Wave 5 | 27.7 (5.0)  (n=1,249) |
